# Real‐world treatment and outcome patterns of patients with mantle cell lymphoma in China: A large, multicenter retrospective analysis

**DOI:** 10.1002/cam4.6009

**Published:** 2023-05-06

**Authors:** Ping Yang, Qing‐qing Cai, Wei Zhang, Shuo‐zi Liu, Hui Liu, Xiu‐hua Sun, Yu‐jun Dong, Xiu‐bin Xiao, Jing‐wen Wang, Zhen‐ling Li, Wen‐rong Huang, Li‐hong Li, Hui‐zheng Bao, Wei Yang, Ya‐lan Wang, Shu‐ye Wang, Juan He, Xiao‐ling Li, Ai‐chun Liu, Hong‐mei Jing

**Affiliations:** ^1^ Department of Hematology Peking University Third Hospital Beijing China; ^2^ Department of Medical Oncology, State Key Laboratory of Oncology in South China, Collaborative Innovation Center of Cancer Medicine Sun Yat‐sen University Cancer Center Guangzhou China; ^3^ Department of Hematology Peking Union Medical College Hospital Beijing China; ^4^ Department of Hematology Beijing Hospital Beijing China; ^5^ Department of Medical Oncology The Second Hospital of Dalian Medical University Dalian China; ^6^ Department of Hematology Peking University First Hospital Beijing China; ^7^ Senior Department of Hematology The 5th Medical Center of PLA General Hospital Beijing China; ^8^ Department of Hematology Beijing Tongren Hospital Beijing China; ^9^ Department of Hematology China‐Japan Friendship Hospital Beijing China; ^10^ Department of Hematology Beijing Tsinghua Changgung Hospital Beijing China; ^11^ Department of Medical Oncology Jilin Cancer Hospital Changchun China; ^12^ Department of Hematology Shengjing Hospital of China Medical University Shenyang China; ^13^ Department of Medical Oncology Baotou Cancer Hospital Baotou China; ^14^ Department of Hematology The First Hospital of Harbin Medical University Harbin China; ^15^ Department of Hematology The First Hospital of China Medical University Shenyang China; ^16^ Department of Medical Oncology Liaoning Cancer Hospital & Institute Shenyang China; ^17^ Department of Hematology and Lymphatic Medicine Harbin Medical University Cancer Hospital Harbin China

**Keywords:** high‐dose cytarabine, maintenance treatment, mantle cell lymphoma, relapse/refractory treatment

## Abstract

**Background:**

Mantle cell lymphoma (MCL) is an uncommon heterogeneous subtype of B cell non‐Hodgkin lymphoma, and clinical features in MCL appear regional characteristics. MCL treatment opinions are not uniform between countries or regions within Asia and China, and Asian patient‐specific data for MCL treatment are fewer. The study aims to explore the clinical characteristics, treatment patterns and prognosis of MCL patients in China.

**Methods:**

A total of 805 patients diagnosed with MCL between April 1999 and December 2019 at 19 comprehensive hospitals in China were included in this retrospective analysis. Kaplan‐Meier method coupled with the log‐rank test was used for univariate analysis, and COX proportional hazards model was used for multivariate analysis (MVA). *p* < 0.05 was consided statistically significant. All outputs were produced using R version 4.1.0.

**Results:**

The median age of the cohort was 60.0 years with a male‐to‐female ratio of 3.36:1. Five‐year progression‐free survival (PFS) and overall survival (OS) rates were 30.9% and 65.0%, respectively. High‐intermediate/high‐risk group according to MIPI‐c, without high‐dose cytarabine, lack of Auto‐SCT as consolidation and maintenance treatment and SD/PD in initial treatment remained statistically relevant to poor PFS on MVA, and ki67 ≥50%, B symptoms, high‐intermediate/high risk group according to MIPI‐c, without high‐dose cytarabine, lack of maintenance treatment, SD/PD in initial treatment and relapse/refractory state were independently associated with poorer OS on MVA.

**Conclusions:**

First‐line high dose cytarabine exposure, auto‐SCT as consolidation therapy obtained survival benefits in Chinese population. Our study further confirmed the value of maintenance treatment and explored the application of new drug treatment and bendamustine in R/R MCL patients.

## 1 INTRODUCTION

Mantle cell lymphoma (MCL) is an uncommon heterogeneous subtype of B‐cell non‐Hodgkin lymphoma, which is characterized by overexpression of cyclin D1 as a result of translocation t (11;14) (q13;q32). MCL comprises 3% to 10% of non‐Hodgkin lymphomas in Western countries.^1^, ^2^, ^3^, ^4^, ^5^ The median age at diagnosis is about 68 years and predominantly occurs in male patients with the ratio of 3:1.^4^, ^6^ Despite the heterogeneity of clinical behaviors and prognosis, most MCL cases have a rapid evolution and an aggressive behavior with an unfavorable outcome.^7^ Median overall survival (OS) following initial induction therapy is 3–5 years with the use of dose‐intense chemotherapy or combination therapy, incorporation of anti‐lymphoma antibodies, and autologous stem cell transplantation (ASCT). Patients with refractory and relapsed disease often respond poorly to chemotherapy and progress rapidly, resulting in the median OS of 1–2 years.^8^

Clinical features in mantle cell lymphoma appeared regional characteristics. The median age of onset was 59–60 years in Asian, which was lower than that reported in European and American countries.^9^, ^10^ The epidemiology of MCL in Asia is not accurate documented but appears to comprise 2%–6% of all lymphoma in different countries. In China, small sample sizes show that the incidence rate varies from 2.6% to 6.3%.^11^, ^12^, ^13^ The standardization of treatment and diagnosis, especially the extensive use of immunohistochemical markers such as CyclinD1 and Sox11, make it helpful to discriminate MCL from other B‐cell lymphomas. Although there is no standard guideline for MCL treatment, the recognized treatment options in young fit patients are aggressive chemo‐immunotherapy containing high‐dose cytarabine combined with autologous hematopoietic stem cell transplantation as the consolidation therapy and rituximab as maintenance therapy. For older patients, chemo‐immunotherapy and rituximab maintenance are commonly used.^9^, ^10^, ^14^, ^15^, ^16^, ^17^ However, MCL treatment opinions are not uniform between countries or regions within Asia and China, and Asian patient‐specific data for MCL treatment are lacking.

As the above background, to address the knowledge gap in Chinese MCL patients, we presented data from a multicenter 20‐year retrospective registry. In this study, we focused on the clinical characteristics, treatment patterns, and prognosis of MCL patients in China.

## 2 MATERIALS AND METHODS

### 2.1 Patients

Patients diagnosed with MCL between April 1999 and December 2019 at 19 comprehensive hospitals in China were included in this retrospective analysis. The median follow‐up duration was 48.0 months. A total of 805 MCL patients were collected, among them, 112 patients with incomplete clinical data, no chemotherapy, and missing follow‐up data were excluded. Finally, 693 patients with complete clinical data and survival follow‐up data were included in the subsequent analysis **(**Figure S1 **)**. Pathological diagnosis was determined according to World Health Organization criteria. Medical records were reviewed for demographic and clinical data. The data included baseline clinical features, physical examinations, biological data, treatment strategies, and survival data. The above medical records and the study protocol conformed to the ethical requirements of Peking University Third Hospital. The study was performed in accordance with the ethical standards of Helsinki and its later amendments.

### 2.2 Data collection

Clinical characteristics of MCL patients included sex, age, the presence of B‐symptoms, Eastern Cooperative Oncology Group (ECOG) performance status, Ann Arbor staging, extranodal disease, blastoid or pleomorphic morphology, Ki‐67 index, blood counts, LDH levels, and β2 microglobulin levels. Treatment information was also collected for analysis, including first‐line chemotherapy, ASCT, maintenance treatment, and treatment options of relapsed/refractory MCL. Risk stratification is mainly based on MIPI score and MIPI‐c score, which were calculated with sufficient data as previously published.

### 2.3 Statistical approach

OS was defined as the date from MCL diagnosis to the date of death from any cause or was censored at the date of last follow‐up for survivors. Progression free survival (PFS) was calculated as the time between the date of diagnosis and the date of relapse, progression, or death from any cause. Fisher's exact test or Pearson's c hi‐squared test were used to compare categorical variables, as appropriate. PFS and OS was estimated according to the Kaplan–Meier survival method. By using univariable and multivariable Cox regression models, comparisons between the variables of interest were conducted. The log‐rank test was then used to determine hazard ratios (HR), the corresponding 95% confidence intervals (95% CI) of mortality and the *p*‐values. Only variables with a certain significance (*p* < 0.05) in the univariable analysis were included in the multivariable model. *p* < 0.05 was considered statistically significant. All outputs were produced using R version 4.1.0.

## 3 RESULTS

### 3.1 Clinical and demographic characteristics

A total of 693 MCL patients with integrated clinical data, treatment, and follow‐up data from 19 centers in China were enrolled in this study. 534 patients were male (77.1%), with a male‐to‐female ratio of 3.36:1. The median age at diagnosis was 60.0 years (range, 25–88 years). There were 478 patients (69.0%) younger than 65 years at diagnosis and 215 patients (31.0%) older than 65 years. Clinical and demographic characteristics of the younger and older patients are summarized in Table 1. Compared with the younger cohort, older patients were more likely to have high ECOG (*p* < 0.001), extranodal organs involvement (*p* = 0.003), bone marrow involvement (*p* = 0.004), and high level of LDH (*p* = 0.044). According to MIPI and MIPI‐c, the proportion of high‐risk group and high‐/high‐intermediate‐risk group in elderly patients is higher (33.5% vs. 11.3% and 58.2% vs. 28.5%, respectively).

**TABLE 1:** Patient demographic and clinical characteristics in MCL.

Characteristics	Young MCL, age <65; *N* = 478	Elderly MCL, age ≥ 65; *N* = 215	*p* value
Age, years, median (range)	55.0 (25–64)	69.0 (65–88)	
Sex			0.649
Male, *n* (%)	366 (76.6%)	168 (78.1%)	
Female, *n* (%)	112 (23.4%)	47 (21.9%)	
Year of diagnosis			0.927
2000–2015, *n* (%)	164 (34.3%)	73 (34.0%)	
2015–2020, *n* (%)	314 (65.7%)	142 (66.0%)	
Pathological subtype			
Blastoid/pleomorphic	66 (13.8%)	23 (10.7%)	0.501
Ann Arbor			0.208
Stage I‐II	43 (9.0%)	26 (12.1%)	
Stage III‐IV	435 (91.0%)	189 (87.9%)	
LDH			0.044
Normal	318 (66.5%)	126 (58.6%)	
High	160 (33.5%)	89 (41.4%)	
B symptoms			0.063
No	328 (68.6%)	132 (61.4%)	
Yes	150 (31.4%)	83 (38.6%)	
ECOG			<0.001
0–1	461 (96.4%)	191 (88.8%)	
≥2	17 (3.60%)	24 (11.2%)	
Ki‐67			0.517
<30%	206 (43.1%)	87 (40.5%)	
≥30%	272 (56.9%)	128 (59.5%)	
Extranodal organ involvement			0.003
No	93 (19.5%)	22 (10.2%)	
Yes	385 (80.5%)	193 (89.8%)	
Bone marrow involvement	203 (42.5%)	117 (54.4%)	0.004
MIPI			<0.001
Low risk	303 (63.4%)	45 (20.9%)	
Intermediate risk	121 (25.3%)	98 (45.6%)	
High risk	54 (11.3%)	72 (33.5%)	
MIPI‐c			<0.001
Low risk	152 (31.8%)	24 (11.2%)	
Low/intermediate risk	190 (39.7%)	66 (30.7%)	
High/intermediate risk	95 (19.9%)	70 (32.6%)	
High risk	41 (8.6%)	55 (25.6%)	
First‐line therapy			
High‐dose Ara‐c	191 (40.0%)	32 (14.8%)	
R‐CHOP	202 (42.3%)	110 (51.2%)	
R‐BTKi±other agents	26 (5.4%)	29 (13.5%)	

We also collected the clinical manifestations of patients at diagnosis. The main symptoms were as follows: superficial tumor or mass (62.6%), abdominal pain or distension (14.1%), dysphagia (7.8%); the second symptoms were fatigue, fever, abnormal hemogram, and hepatosplenomegaly.

### 3.2 First‐line and maintenance treatment

In our study, all 693 patients received chemotherapy as first‐line treatment. The therapeutic schedule was not completely unified. Among them, the most frequently regimen is CHOP/CHOP‐like±R with 312 patients (45.0%) used. Besides, 222 patients(32.0%)were treated with high‐dose cytarabine, 154 patients(22.2%) received CHOP / DHAP±R regimen, 45 patients (6.5%) received dose adjusted hyper CVAD±R regimen, 23 patients (3.3%) received high cytarabine + R regimen, 44 patients (6.3%) received VR‐CAP regimen, 30 patients (4.3%) received BR regimen, 55 patients(7.9%) initially chose chemo‐free regimen including IR/R2/IR2, and other less used initial treatments included R‐EPOCH (*n* = 17, 2.5%) and FC/FCM ± R regimen (*n* = 13, 1.9%) **(**Figure S2 **)**. Despite the high proportion of young patients, only 80 patients (11.5%) received autologous hematopoietic stem cell transplantation as consolidation therapy after chemotherapy remission.

In this study, 309 patients (44.6%) received maintenance therapy as consolidation treatment after initial treatment. Among them, 151 patients (21.8%) received rituximab maintenance regimen, 43 patients (6.2%) received lenalidomide maintenance regimen, 47 patients (6.8%) received ibrutinib maintenance therapy, and 67 patients (9.7%) received IR/R2 regimen as the maintenance therapy.

### 3.3 Response data and relapse/refractory treatment

In the initial treatment, the overall response rate (ORR) was 85.0%, with 46.6% of complete remission (CR) rate and 38.4% of partial remission (PR) rate. Furthermore, the study compared the efficiency between different first‐line treatment regimens. The results showed that the ORR rate and CR rate in high‐dose cytarabine regimen were better than other non high‐intensity treatment regimens (92.4% vs. 81.5%, *p* < 0.001and 68.6% vs. 36.2%, *p* < 0.001, respectively). In terms of treatment‐related mortality, intensify chemotherapy was slightly higher than the non‐intensify group (4.1% vs. 3.3%, *p* = 0.167). The main cause was related to serious infection and multiple organ failure.

During the follow‐up time, 409 (59.0%) patients were progressed to relapsed or refractory cases, of which 104 patients (15.0%) become refractory and 305 patients (44.0%) relapsed after remission. We further analyzed the correlation between clinical parameters and relapsed/refractory MCL patients. Binary logistic regression analysis showed that age ≥65 years, stage III/IV by Ann Arbor staging, B symptoms, high‐risk group according to MIPI‐c and MIPI index, elevated LDH level, and initial treatment without high‐dose Ara C and without maintenance treatment were the related factors of replased/refractory MCL patients. Multiple logistic regression analysis suggested that elevated LDH level and initial treatment without high‐dose Ara C and without maintenance treatment were the independent related factors in relapse/refractory MCL.

Among them, 360 available patients received treatment including salvage chemotherapy, new drug therapy, and BR regimen chemotherapy. The new drugs mainly included lenalidomide, ibrutinib, and bortezomib, which were used alone or in combination with other drugs. Among these 360 patients, 125 patients received salvage chemotherapy only, and the ORR was 29.6% and CR was 7.2%; 205 patients received new drugs single or combined treatment with 64.4% of ORR and 16.1% of CR; 30 patients were treated with BR regimen, and the ORR was 53.3% and CR was 23.3%, respectively.

### 3.4 Survival and prognostic factors

**FIGURE 1:**
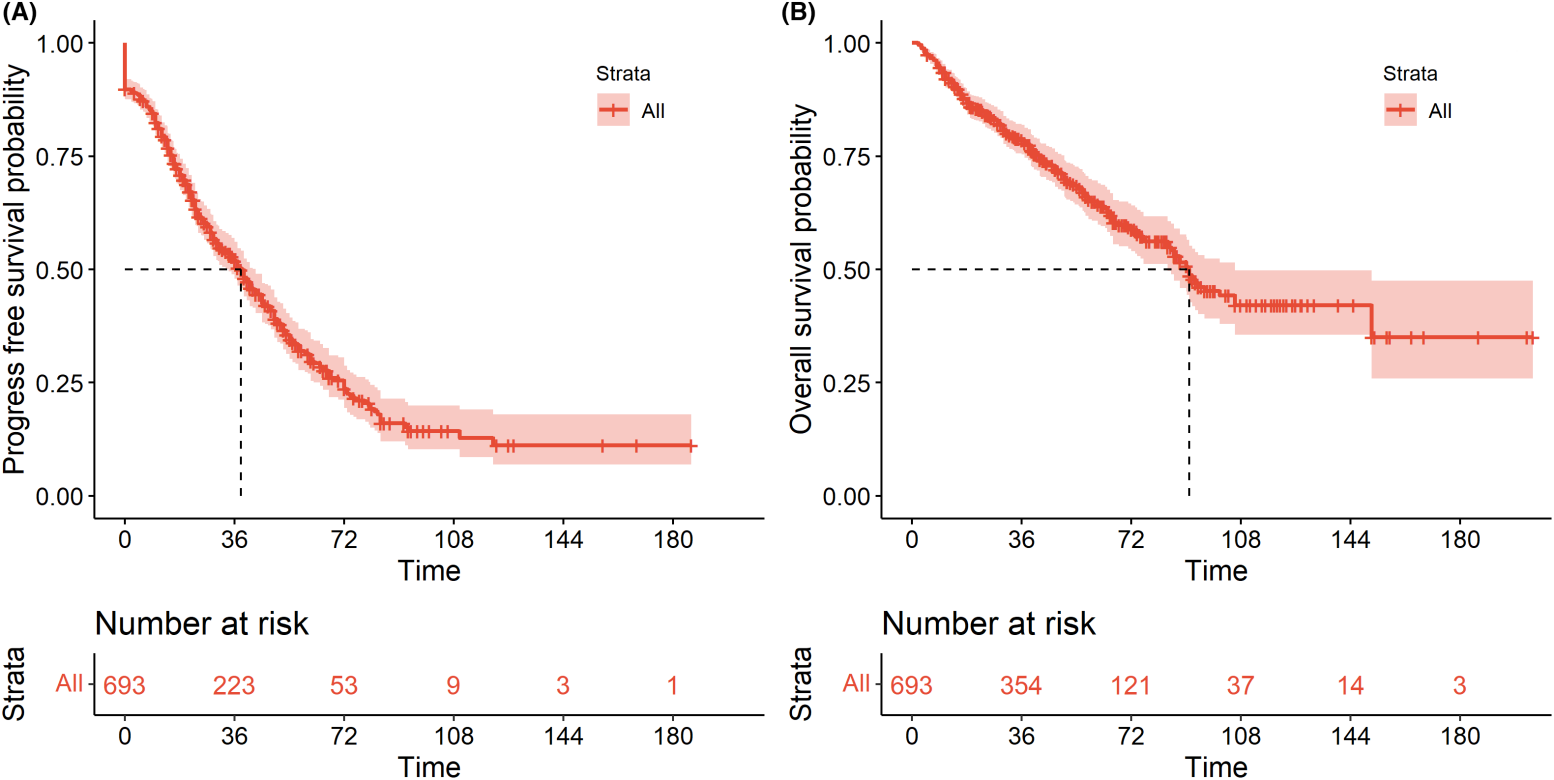
Outcomes of 693 patients with mantle cell lymphoma. (A) Progression‐free survival. (B) Overall survival.

During the follow‐up to June 2021, 222 patients (32.0%) died. The 3‐year and 5‐year PFS of the global series was 51.5% and 30.9%, and the 3‐year and 5‐year OS was 78.6% and 65.0%, respectively (Figure 1). In univariable analysis, we analyzed the common variables, including age, gender, tumor proliferation index, pathological type, stage, B symptoms, LDH level, ECOG, extranodal organ involvement, different extranodal involvement sites, MIPI index, and MIPI‐c index. At the same time, we also analyzed the treatment related factors, including initial treatment regimens, autologous hematopoietic stem cell transplantation, initial therapeutic efficacy, relapsed/refractory and treatment lines (Tables S1 and S2).

The result showed that age ≥65 years, ki67 ≥30%, ki67 ≥ 50%, stage III‐IV, B symptoms, elevated LDH, extranodal organ involvement, spleen involvement, bone marrow involvement, high‐risk group according to MIPI/MIPI‐c index, high‐dose cytarabine in first‐line therapy, auto‐ASCT, maintenance treatment, CR/PR in initial treatment were significantly correlated with worse PFS in univariable analysis. Except of the above factors, year of diagnosis, pathological subtype and relapsed/refractory state were associated with poor OS in univariable analysis (Tables S1 and S2).

Low‐intermediate‐/low‐risk group according to MIPI‐c, high‐dose cytarabine regimen, auto‐ASCT as consolidation therapy, CR/PR after initial treatment, and maintenance treatment were associated with better PFS on multivariable analyses (Figure S3). Meanwhile, ki67 ≥50%, B symptoms, high‐intermediate‐/high‐risk group according to MIPI‐c, without high‐dose cytarabine, lack of maintenance treatment, CR/PR after initial treatment and relapse/refractory state were the independent prognostic factors for OS (Figure S3).

**FIGURE 2:**
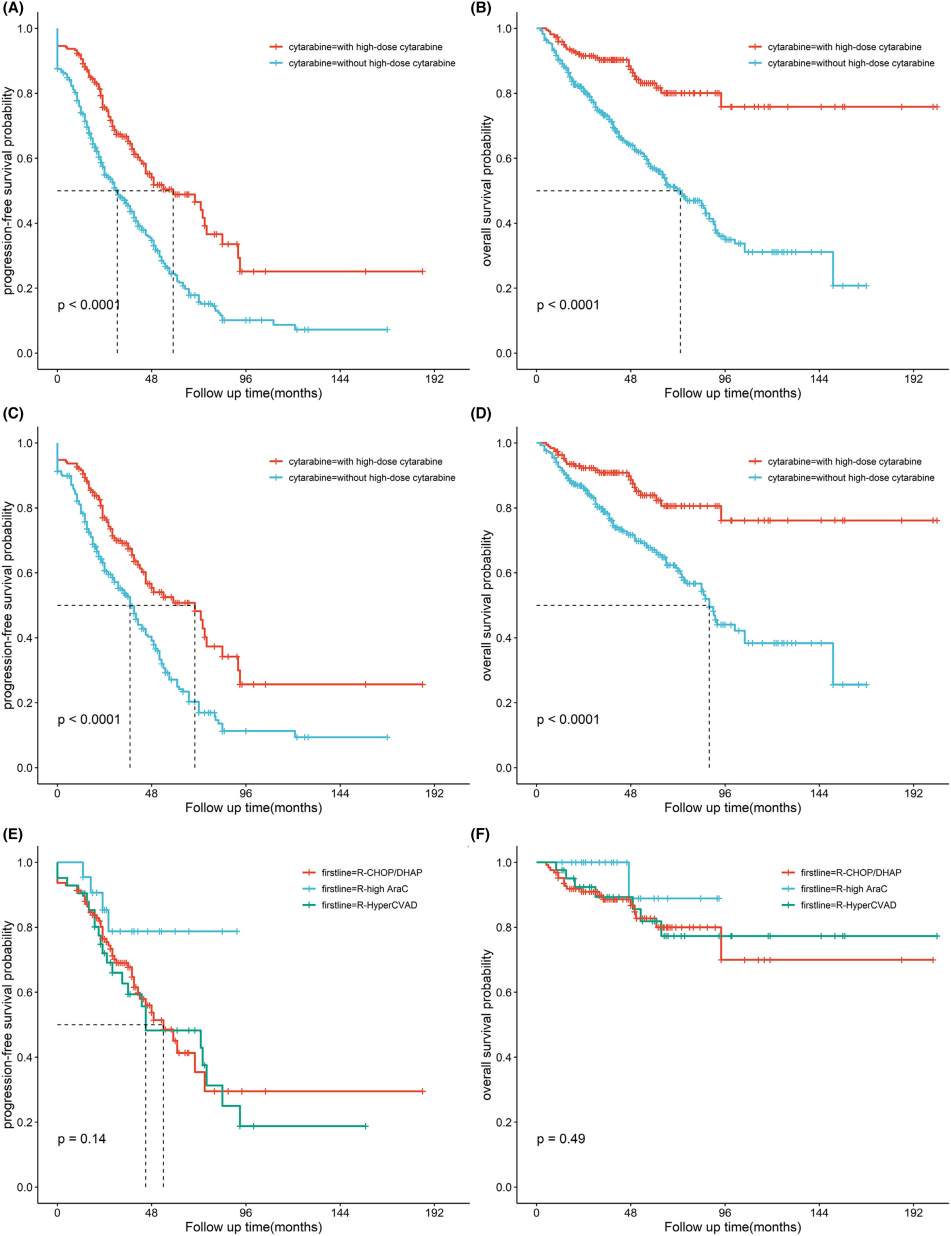
Progression‐free survival (A) and overall survival (B) of patients with mantle cell lymphoma differed according to use of high‐dose Arac regimens; In younger patients (age < 65), use of high‐dose Arac treatment showed a trend for improved PFS (C) and improved OS (D); different high‐dose cytarabine regimens show no significant difference in PFS (E) and OS (F) in younger patients.

In our cohort, we compared PFS and OS according to different induction regimens. There were significant differences in PFS and OS between high‐dose cytarabine‐containing regimen group and non‐intensified chemotherapy group, and the 5 year‐PFS and OS were 48.9% versus 24.0% and 81.7% versus 57.0%, respectively (*p* < 0.001, *p* < 0.001, Figure 2). Among the young patients, 191 patients chose the treatment regimen containing high‐dose cytarabine in the initial treatment. As same as the overall population, the high‐dose cytarabine treatment group has the survival advantage in the young cohort, with the 5 year‐PFS and OS were 50.4% versus 27.3% and 83.2% versus 67.5%, respectively (*p* < 0.001, *p* < 0.001, Figure 2). Further, we compared the different cytarabine‐containing regimens in the young cohort. Among them, 122 young patients received CHOP/DHAP±R regimen, 42 patients received dose adjusted hyper CVAD±R regimen, and 22 patients received R‐ high cytarabine regimen. There was no significant difference in PFS and OS between different high‐dose cytarabine regimens (*p* = 0.144, *p* = 0.494, Figure 2).

55 patients (7.9%) initially chose chemo‐free regimen including IR/R2/IR2 due to unfit condition or refusing chemotherapy. The ORR rate and CR rate were 81.8% and 23.6% in this group, and the 5‐year PFS and 5‐year OS were 34.5% and 57.1%, respectively. There was no significant difference in PFS when compared with chemo‐regimens (*p* = 0.359), however, chemo‐regimens was better than chemo‐free group in OS (*p* = 0.003), which may due to the unfit state and short follow‐up time in chemo‐free cohort.

**FIGURE 3:**
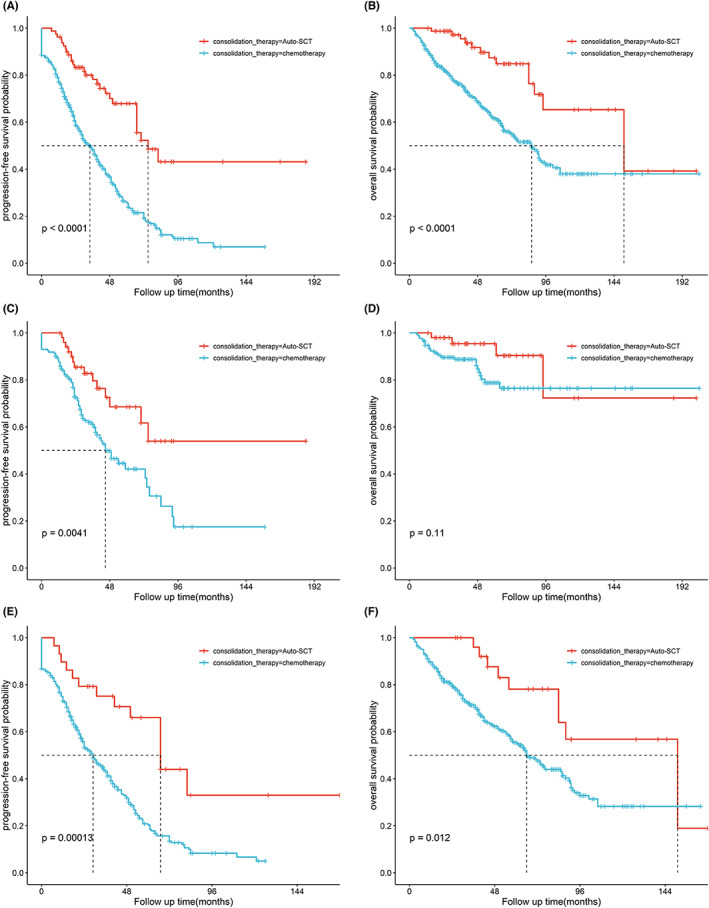
Progression‐free survival (A) and overall survival (B) of MCL patients with the usage of Auto‐SCT as consolidation therapy; induction with high‐dose cytarabine, auto‐SCT as consolidation therapy showed improved PFS (C) and no significant difference in OS (D); induction with non‐intensive chemotherapy, auto‐SCT as consolidation therapy had a significant improvement in PFS (E) and OS (F).

Survival was compared in patients who were treated with ASCT (*N* = 80) and non‐ASCT as consolidation therapy (Figure 3). The 5‐year PFS rates were 68.8% versus 25.3% (*p* < 0.001) and the 5‐year OS rates were 87.3%versus 61.7% (*p* < 0.001), respectively. We further compared PFS and OS according to induction regimens with or without ASCT. Among non‐intensified induction regimens with ASCT (*n* = 29) and without ASCT (*n* = 441), the 5‐year PFS and 5‐year OS rate were 66.0% versus 20.3% (*p* < 0.001), and 78.1% versus 55.4% (*p* = 0.012), respectively (Figure 3). In high‐dose cytarabine induction regimens with ASCT (*n* = 51) and without ASCT (*n* = 172), the 5‐year PFS and 5‐year OS rate were 68.5% versus 42.1% (*p* = 0.004) and 90.4% versus 78.8% (*p* = 0.11), respectively (Figure 3).

**FIGURE 4:**
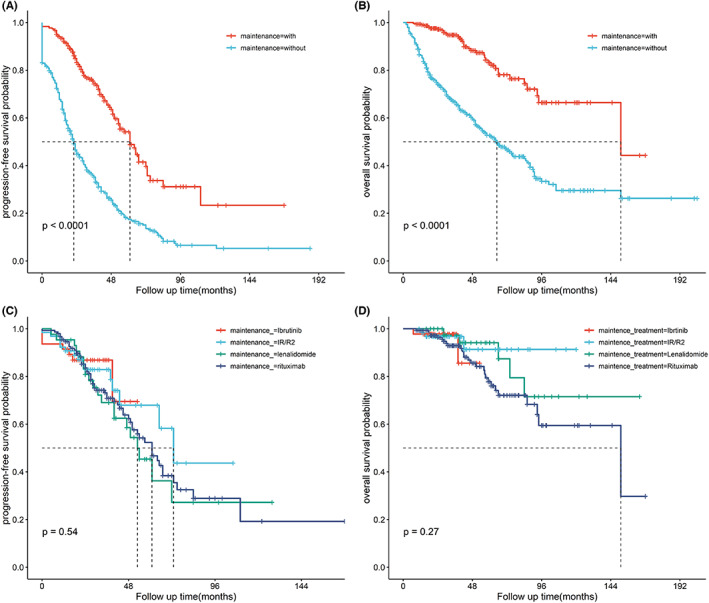
Progression‐free survival (A) and overall survival (B) of MCL patients with maintenance treatment. There is no significant difference between different maintenance therapy including rituximab, lenalidomide, ibrutinib and IR/R2 regimens in PFS (C) and OS (D).

Maintenance regimen in our research was not consistent. We further compared PFS and OS with or without MR. The 5‐year PFS and 5‐year OS rate in the maintenance regimen and non‐maintenance regimen group were 53.6% versus 17.2% (*p* < 0.001), and 82.7% versus 52.9% (*p* < 0.001), respectively. However, there was no significant difference in PFS (*p* = 0.520) and OS (*p* = 0.270) between different maintenance therapy including rituximab, lenalidomide, ibrutinib, and IR/R2 regimens (Figure 4).

Progression of disease (POD) within 24 months was considered an indicator of poor prognosis in indolent lymphoma. In this study, we compared with POD24 and non POD24, and there was a statistical difference between the two group in OS (*p* < 0.0001; Figure S4). The median OS time was 151 m versus 32 m in POD24 and non POD24 group, respectively.

The Kaplan–Meier method was applied to estimate time‐to‐event outcomes (OS‐2) and comparisons between treatment groups were performed in our cohort. Compared with salvage chemotherapy, new drugs treatment and BR regimen had obvious survival advantages and the median OS‐2 for patients with new drugs treatment and BR regimen were 21.0 and 23.0 months respectively, which were longer than that treated with salvage chemotherapy (*p* < 0.001, Figure S5).

### 3.5 Second neoplasms

During the follow‐up period, 34 patients (4.90%) complicated with or developed a secondary malignancy. As Table S2 shown, the most common secondary malignancy was lung cancer (*n* = 7). The second was hematological malignancies, including AML, ALL, MDS, and DLBCL (*n* = 6), followed by breast cancer (*n* = 4), prostate cancer (*n* = 3), gastric cancer (*n* = 3), and colon cancer (*n* = 3).

## 4 DISCUSSION

MCL is a rare B‐cell lymphoma that is described as an aggressive, generally incurable lymphoma with formerly poor long‐term survival.^1^, ^2^, ^3^ Previous reports have shown that the incidence rate of MCL in Asia was significantly lower than that in western countries.^9^, ^10^ Based on the observational database research study, MCL accounted for approximately 3% of malignant lymphoma in Japan.^18^ To the best of our knowledge, this was the largest retrospective study limited to MCL in Chinese population. In terms of demographic characteristics, the number of patients in this study increased significantly after 2015, which was mainly related to the improvement in diagnostic level, especially the immunohistochemical markers of CyclinD1 and Sox11 that were used as routine examinations for the diagnosis of B‐cell lymphoma in large hospitals in China. MCL often occurs in male patients in the Asian population, and the median age at initial presentation is 60 years, which is significantly lower than that in Western and American countries. In pathological subtypes, compared with previous reports, 10%–15% of patients presented with a more indolent subtype and non‐nodular MCL only accounted for 3.3% in our cohort and the majority of these patients have symptoms and treatment indications.^19^ Most of the newly diagnosed patients displayed aggressive disease features, among which blastic/pleomorphic mantle cell lymphoma accounted for 12.8%, ki‐67≥30% accounted for 57.7% and ki‐67≥50% accounted for 26.1%. Meanwhile, the proportion of extranodal involvement was higher, accounting for 83.4% in this patient cohort.

The standard frontline therapy for MCL is not completely unified, however, there is a general consensus that cytarabine‐containing chemotherapy combined with ASCT as the first‐line treatment for young and fit patients, whereas older or unfit patients are treated with combination chemo‐immunotherapy according to different tolerance.^9^, ^20^, ^21^, ^22^, ^23^ Although the benefit of high‐dose cytarabine is clear, the recognized unified regimen is not definite due to different clinical trial results. In European countries, dose‐intensified with cytarabine‐containing regimen is recommended as induction therapy. The Nordic group (MCL2) and French study (GELA) evaluated the efficacy of high‐dose cytarabine, and the European MCL Network confirmed the superiority of R‐CHOP/R‐DHAP followed by ASCT which recommended as the standard induction regimen in younger MCL patients.^24^, ^25^, ^26^, ^27^ In the USA, MD Anderson Cancer Center pioneered the regimen of R‐hyper CVAD which established efficacy in younger patients and was obtained from two other large cohorts. Long‐term follow‐up confirmed the efficacy of Hyper CVAD as a frontline regimen for younger patients.^28^, ^29^, ^30^, ^31^ For young MCL patients in Asia, although experts agreement and consensus had recommended cytarabine‐containing as the first‐line regimen, there is no unified proposal for the concrete scheme.^10^ In our study, 127 patients used the R‐CHOP/DHAP regimen, 22 patients chose high‐dose Ara C regimen, and 42 patients used dose‐adjusted R‐hyper CVAD regimen as induction therapy. There was no significant difference in PFS and OS between different high‐dose cytarabine regimens. However, hampered by severe hematological toxicity, R‐CHOP/R‐DHAP is a probable better choice for Asian MCL patients in terms of therapy‐associated tolerance compared with R‐hyper CVAD regimen. Apart from the standard treatment options discussed above, the combined regimens containing new drugs such as BTKi or lenalidomide have been used in clinical trials.^32^, ^33^ Chemotherapy‐free treatment is effective in indolent lymphoma and the usage in MCL as first‐line is still in the process of clinical trials.^3^, ^34^ In this cohort, 55 patients had chemo‐free therapy including IR/R2/IR2 as the initial treatment. There was no significant survival advantage compared with the chemo‐regimen, mainly because this population was elderly and unfit with the short follow‐up time and lack of parallel control.

Although the role of ASCT as consolidation therapy in MCL is still controversial, large‐scale studies have proved that ASCT can significantly improve PFS in younger, transplantation‐eligible patients with MCL.^15^, ^26^, ^35^, ^36^, ^37^ In our study, the lower transplantation utilization rate in MCL was due to comprehensive factors such as age, region, treatment safety, and socioeconomic status, which was similar to the recent report about auto‐SCT activity for lymphoma in China^38^ However, ASCT consolidation after induction was associated with significantly improved PFS and OS. Further stratification demonstrated that ASCT after high‐dose cytarabine as induction treatment could improve PFS, but had no significant benefit on OS, while the non‐intensive treatment induction could benefit significantly in PFS and OS. The lack of improvement in OS after dose‐intensified regimens may be a result of effective salvage therapy (e.g., various novel agents and/or CD19‐CART) after relapse, which may abrogate any improvement of consolidative ASCT after induction.

Maintenance treatment following ASCT or induction therapy was considered standard of care for MCL patients. Based on the results of the large‐scale trials, rituximab maintenance had been confirmed a significantly optimization of PFS and OS in young or elderly MCL patients.^39^, ^40^ Recently, FIL study revealed a benefit from lenalidomide maintenance after autologous transplantation with improved PFS, highlighting the role of lenalidomide maintenance in MCL.^41^ The role of ibrutinib in MCL maintenance treatment is in the process of clinical trials, the largest of which is the phase 3 trial Tringle study conducted by the European MCL working group. In the consensus of the Asian Lymphoma Study Group, the choice of maintenance approach should be individualized, with cost being an important consideration.^10^ In our cohort, the maintenance scheme was not consistent due to affordability and reimbursement status. Among our cohort, 44.6% patients received maintenance treatment including rituximab or lenalidomide or BTKi single or combined together. Among these patients, 21.8% patients received rituximab maintenance therapy. Despite the fact that the majority of patients did not receive sufficient maintenance treatment time, it has been verified the efficacy of maintenance therapy in Asian patient populations. Statistics did not demonstrate the difference between different maintenance schemes, the prospective clinical trial is needed to explore the optimum maintenance scheme in the future.

The genetic instability of MCL brings about inevitable replase. Similar to previous reports, 59.0% of patients in this cohort were relapsed/refractory MCL, of which 44.6% patients were relapsed after remission. In evaluating the replased/refractory MCL, we demonstrated that elevated LDH level, initial dose‐intensified therapy and maintenance treatment had significant prognostic value. Treatment choice at relapse represented a unique challenge that was dependent on various patient factors, prior therapy and remission duration. Novel cellular therapies and immunotherapies were currently evaluated in the relapsed/refractory MCL. New drugs including bortezomib, lenalidomide, ibrutinib and the new generation of BTK inhibitors zanubrutinib, orelabrutinib, venetoclax, have been applied as single agent or in combination with immunochemotherapies or other targeted therapies in China.^42^, ^43^, ^44^, ^45^, ^46^, ^47^, ^48^, ^49^, ^50^ BTK inhibitors were recognized as an effective treatment strategy in R/R MCL and BTKi‐based combination were evaluated by prospective trials. Bortezomib and lenalidomide were all therapeutic options before the covalent BTKi era. There are no published or ongoing clinical trials comparing BTKi monotherapy with immunochemotherapy in R/R MCL. In our study, we analyzed the remission and survival of R/R MCL patients treated with different treatment regimens in the real‐word. Similar to the results of MANTLE‐FIRST study,^51^ patients treated with new drugs and bendamustine improved the overall survival (OS‐2) than previous second‐line/third‐line chemotherapy, and no significant differences were observed between new drugs and bendamustine treatments. Due to the limitation of retrospective analysis, the usage of new drugs in R/R MCL patients was diverse, such as single or combination with other targeted /chemotherapy drugs. We cannot accurately evaluates the survival benefit of the single new drug. Despite the high recurrence rate, the survival of Chinese patients with MCL has improved compared with previous reports. POD24 is a clinically relevant endpoint for identifying high‐risk and poor prognoses in patients with follicular lymphoma.^52^ Although few studies have been performed, early POD is associated with inferior survival after intensive or less intensive front‐line therapy of MCL.^25^, ^53^ In our study, POD24 was confirmed as an important prognostic factor in MCL. The usage of BTKi and short follow‐up time in this study are still insufficient. The effect of new drug treatment on POD24 still needs further research.

This study is a large population‐based study of MCL patients from Chinese population diagnosed over 20 years, with access to clinical data, detailed treatment, survival analysis and relapsed/refractory selection. In summary, our study described the demographic characteristics and patient‐specific data for the treatment of MCL in China, revealing the survival benefits of initial dose‐intensified therapy and ASCT in young Chinese patients. Further, we confirmed the value of maintenance treatment and explored the application of new drug treatment and bendamustine in R/R MCL patients. Although not included in this retrospective analysis, we also pay attention to the progress of molecular biology in MCL and individualized guidance, and clinical trials of new drugs in the first‐line application. The treatment mode in MCL may be further changed in the future.

## AUTHOR CONTRIBUTIONS

**Ping Yang:** Conceptualization (equal); formal analysis (equal); writing – review and editing (equal). **Qing‐Qing Cai:** Conceptualization (equal); writing – review and editing (equal). **Wei Zhang:** Conceptualization (equal); writing – review and editing (equal). **Shuo‐zi Liu:** Formal analysis (equal); writing – review and editing (equal). **Hui Liu:** Writing – review and editing (equal). **Xiu‐hua Sun:** Writing – review and editing (equal). **Yu‐jun Dong:** Writing – review and editing (equal). **Xiu‐bin Xiao:** Writing – review and editing (equal). **Jing‐wen Wang:** Writing – review and editing (equal). **Zhen‐ling Li:** Writing – review and editing (equal). **Wen‐rong Huang:** Writing – review and editing (equal). **Li‐hong Li:** Writing – review and editing (equal). **Hui‐zheng Bao:** Writing – review and editing (equal). **Wei Yang:** Writing – review and editing (equal). **Ya‐lan Wang:** Writing – review and editing (equal). **shu‐ye wang:** Writing – review and editing (equal). **Juan He:** Writing – review and editing (equal). **Xiao‐ling Li:** Writing – review and editing (equal). **Ai‐chun Liu:** Writing – review and editing (equal). **Hong‐mei Jing:** Writing – original draft (equal); writing – review and editing (equal).

## FUNDING INFORMATION

This work was funded by Wu Jieping Medical Foundation (320.6750) and key funds in Peking University Third Hospital (BYSYDL2021006). Employees of the funding source were involved in the collection and assembly of data, performing statistical analysis, analyzing and interpreting data, and drafting, reviewing, and approving the manuscript, as reflected in the author contributions statement.

## CONFLICT OF INTEREST STATEMENT

The authors declare that they have no competing interests.

## ETHICS STATEMENT

This study was authorized by The Ethics Committee of Peking University Third Hospital. Informed consensus was obtained from all patients.

## Supporting information


Supplemental Figure 1.

Supplemental Figure 2.

Supplemental Figure 3.

Supplemental Figure 4.

Supplemental Figure 5.

**SUPPLEMENTAL TABLE 1** Pre‐treatment Factors Associated with PFS and OS in MCL patients on univariate COX analysis
**SUPPLEMENTAL Table 2** Post‐treatment Factors Associated with PFS and OS in MCL patients on univariate COX analysis
**SUPPLEMENTAL Table 3** The proportion of combined and secondary tumors in MCL patients

## Data Availability

All supporting data are included in the manuscript. Additional data are available upon reasonable request to the corresponding author.
