# Supplementary figures and images for: Real‐world treatment and outcome patterns of patients with mantle cell lymphoma in China: A large, multicenter retrospective analysis
Source: Cancer Med. 2023 May 6;12(12):13204–16. doi: 10.1002/cam4.6009 (PMC10315753; doi:10.1002/cam4.6009)

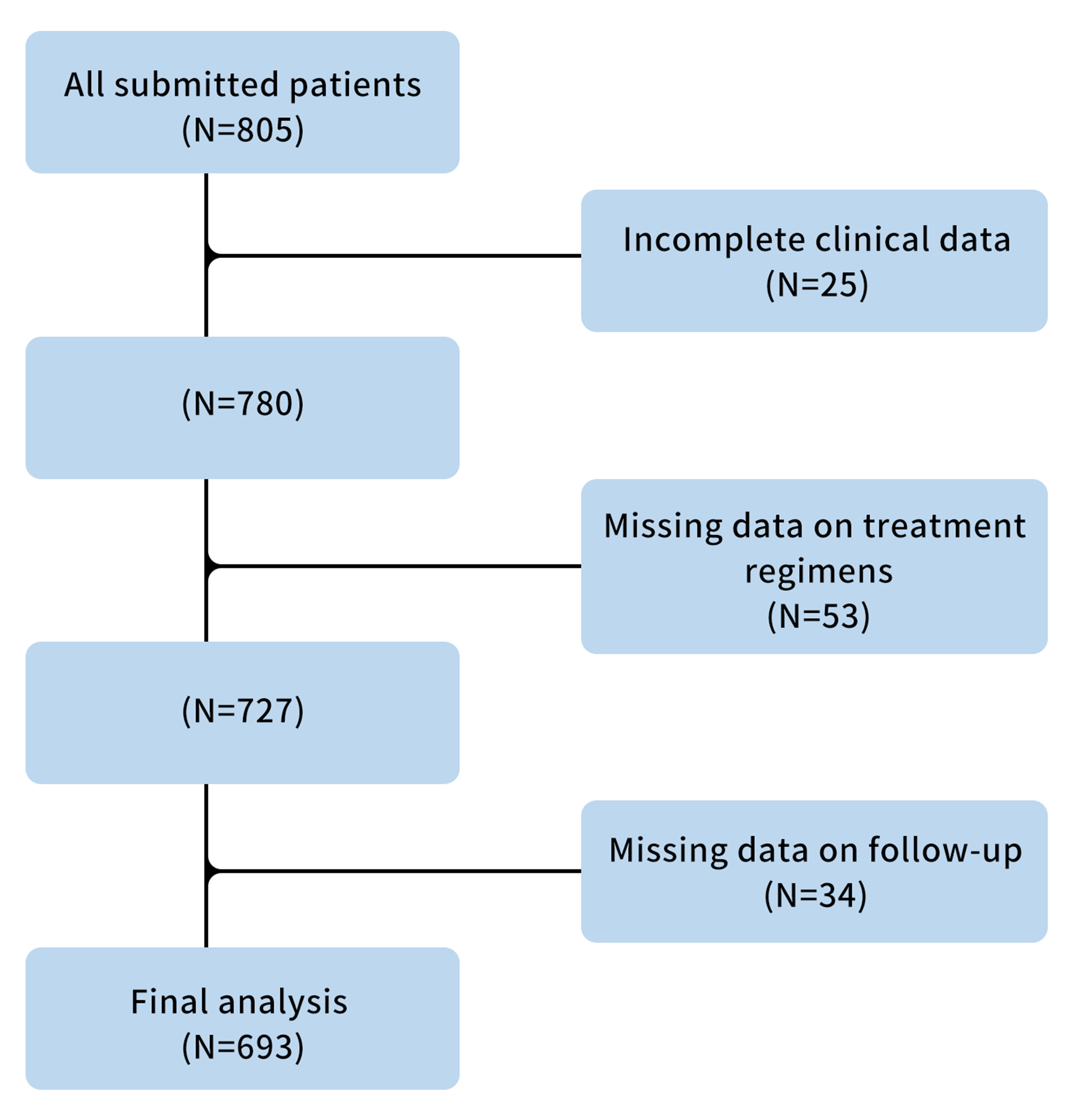

Supplement: Supplementary file 1 — Supplemental Figure 1. [file CAM4-12-13204-s003.tif]

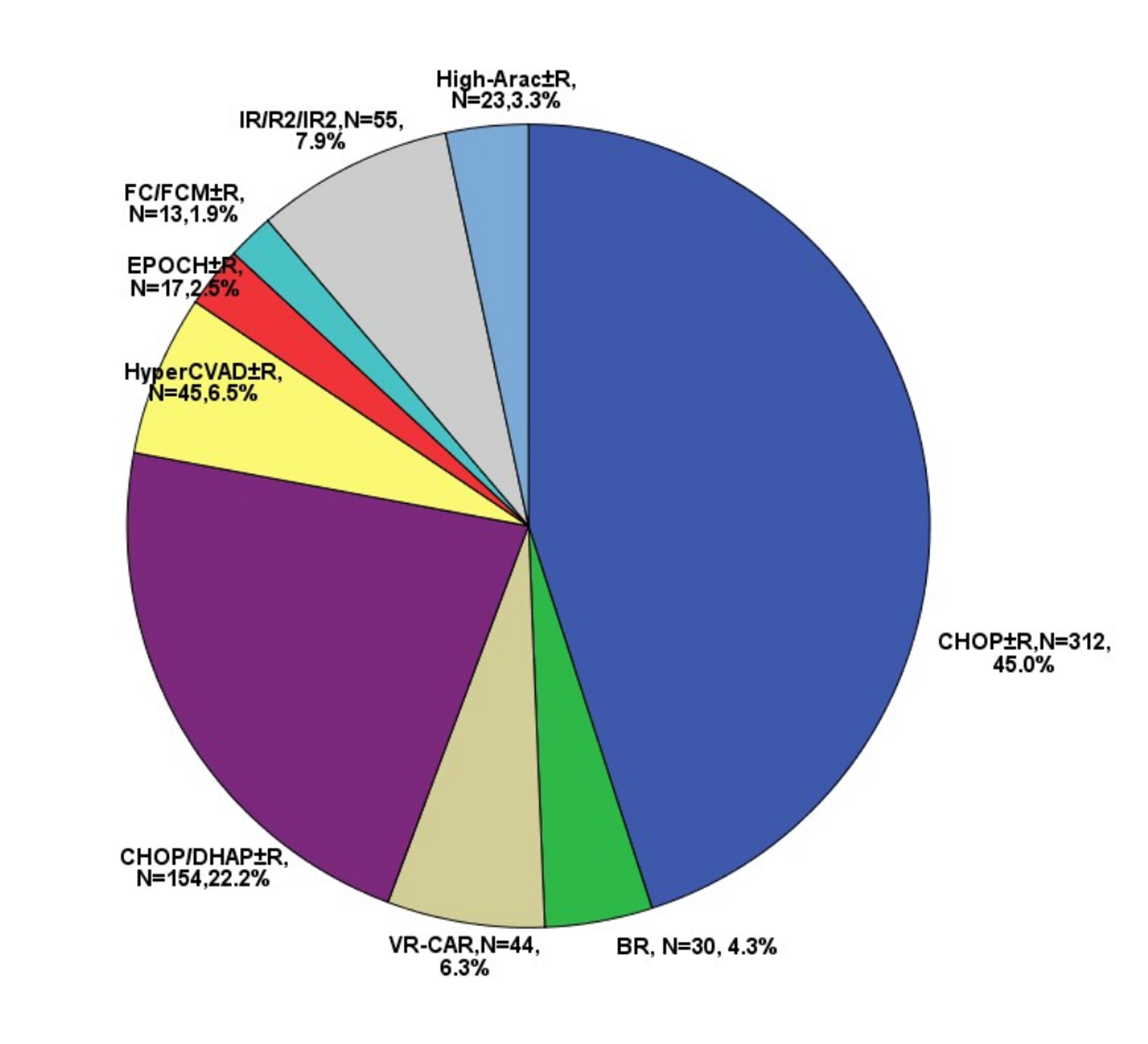

Supplement: Supplementary file 2 — Supplemental Figure 2. [file CAM4-12-13204-s002.tif]

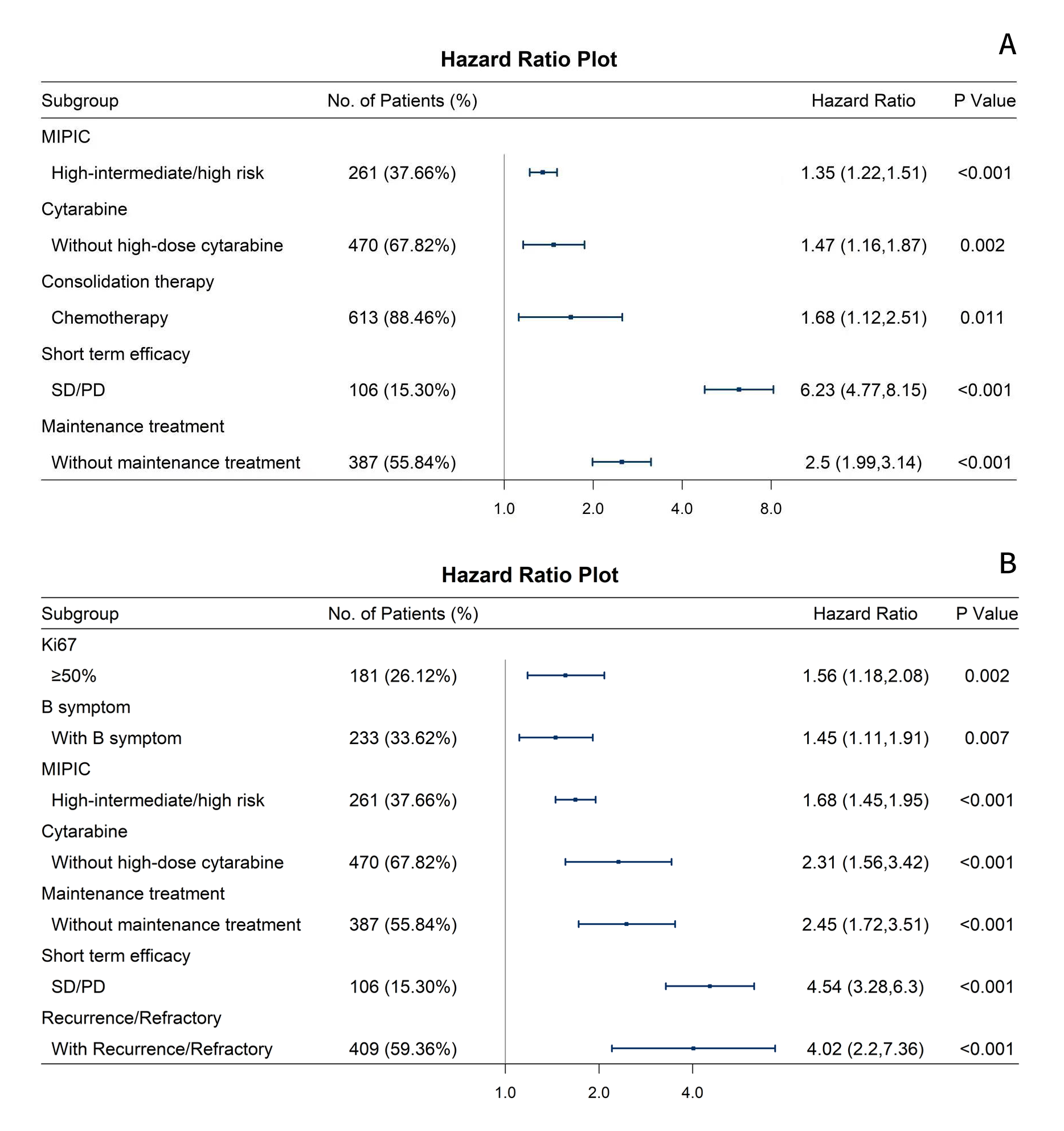

Supplement: Supplementary file 3 — Supplemental Figure 3. [file CAM4-12-13204-s001.tif]

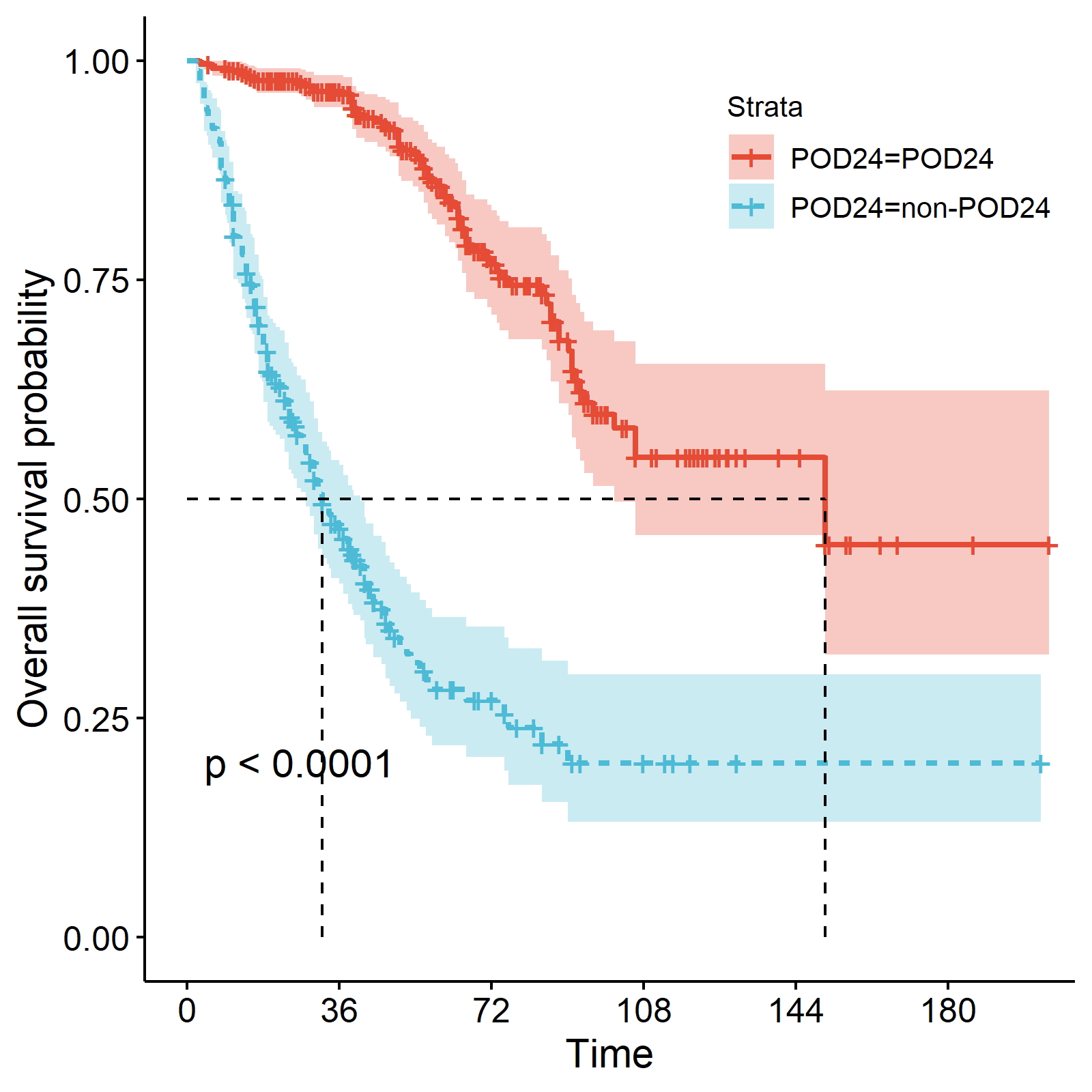

Supplement: Supplementary file 4 — Supplemental Figure 4. [file CAM4-12-13204-s004.tiff]

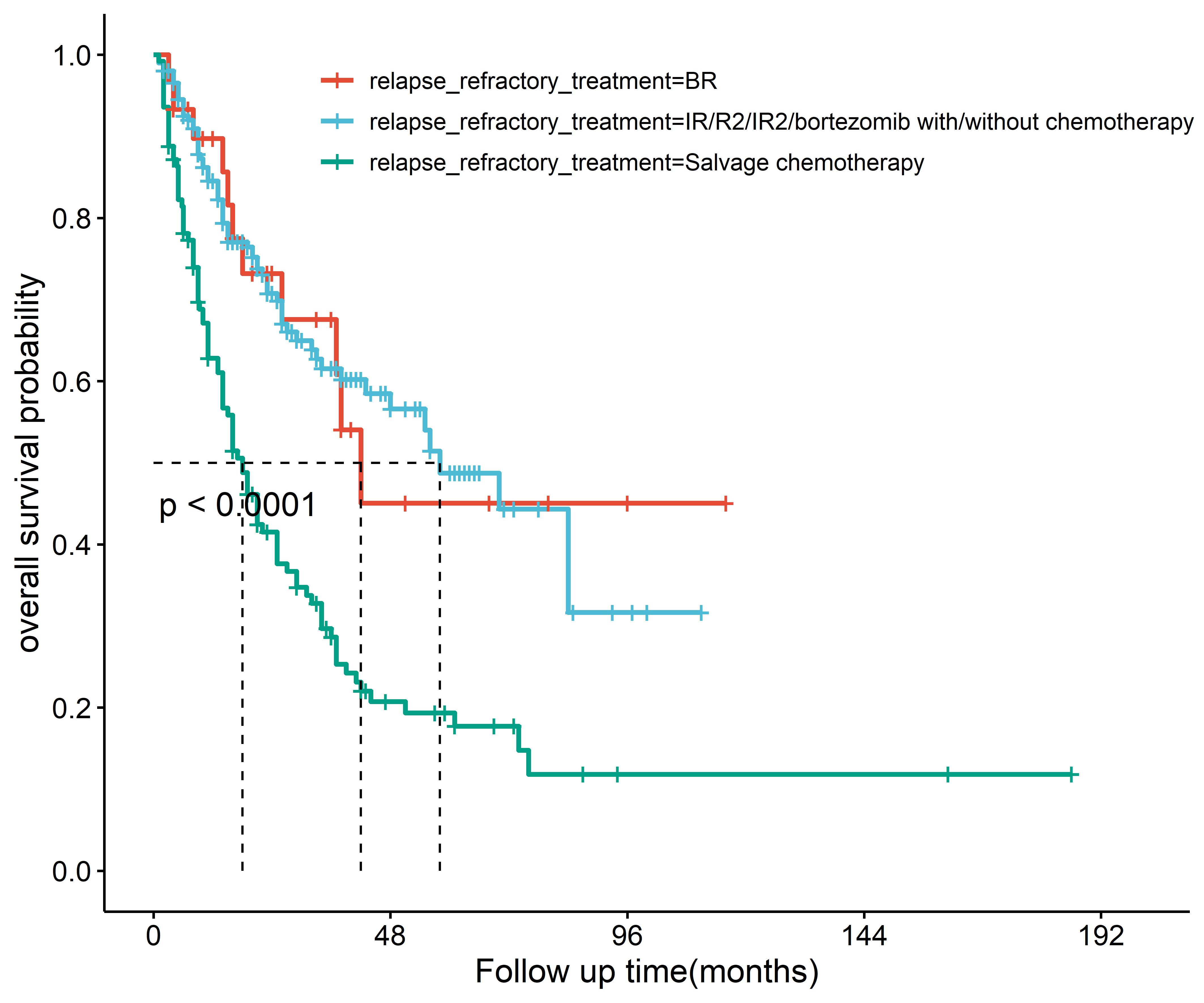

Supplement: Supplementary file 5 — Supplemental Figure 5. [file CAM4-12-13204-s005.tiff]
