# Supplementary material for: Real‐world treatment and outcome patterns of patients with mantle cell lymphoma in China: A large, multicenter retrospective analysis
Source: Cancer Med. 2023 May 6;12(12):13204–16. doi: 10.1002/cam4.6009 (PMC10315753; doi:10.1002/cam4.6009)
Supplement: Supplementary file 6 — SUPPLEMENTAL TABLE 1 Pre‐treatment Factors Associated with PFS and OS in MCL patients on univariate COX analysis SUPPLEMENTAL Table 2 Post‐treatment Factors Associated with PFS and OS in MCL patients on univariate COX analysis SUPPLEMENTAL Table 3 The proportion of combined and secondary tumors in MCL patients [file CAM4-12-13204-s006.docx]

**SUPPLEMENTAL TABLE 1** Pre-treatment Factors Associated with PFS and OS in MCL patients on univariate COX analysis

| **Variable** | | **N (%)** | **OS HR (95%CI,P value)** | **PFS HR (95%CI,P value)** |
| --- | --- | --- | --- | --- |
| Age | <65 | 478 (69.0%) |  |  |
|  | ≥65 | 215 (31.0%) | 2.31 (1.76-3.03, p<.001) | 1.66 (1.35-2.03, p<.001) |
| Ki67(30%) | ＜30% | 293 (42.3%) |  |  |
|  | ≥30% | 400 (57.7%) | 1.81 (1.37-2.40, p<.001) | 1.34 (1.10-1.64, p=0.004) |
| Ki67(50%) | <50% | 512 (73.9%) |  |  |
|  | ≥50% | 181 (26.1%) | 2.04 (1.55-2.69, p<.001) | 1.29 (1.04-1.61, p=0.022) |
| Subtype | Blastoid/Pleomorphic | 89 (12.8%) |  |  |
|  | Classical | 581 (83.8%) | 0.66 (0.45-0.98, p=0.038) | 0.82 (0.61-1.10, p=0.191) |
|  | Indolent type | 23 (3.3%) | 1.07 (0.52-2.20, p=0.847) | 0.99 (0.55-1.79, p=0.967) |
| Stage | I-II | 69 (10.0%) |  |  |
|  | III-IV | 624 (90.0%) | 2.25 (1.25-4.02, p=0.006) | 1.68 (1.17-2.40, p=0.005) |
| B symptom | No | 460 (66.4%) |  |  |
|  | Yes | 233 (33.6%) | 1.81 (1.39-2.36, p<.001) | 1.38 (1.13-1.70, p=0.002) |
| MIPIC | High-intermediate/high risk | 261 (37.7%) |  |  |
|  | Low-intermediate/low risk | 432 (62.3%) | 0.29 (0.22-0.38, p<.001) | 0.46 (0.38-0.56, p<.001) |
| LDH | Elevated | 249 (35.9%) |  |  |
|  | Normal | 444 (64.1%) | 0.42 (0.32-0.55, p<.001) | 0.55 (0.45-0.67, p<.001) |
| spleen involved | No | 443 (63.9%) |  |  |
|  | yes | 250 (36.1%) | 1.63 (1.25-2.12, p<.001) | 1.28 (1.05-1.56, p=0.015) |
| BM involved | No | 373 (53.8%) |  |  |
|  | Yes | 320 (46.2%) | 2.22 (1.69-2.91, p<.001) | 1.33 (1.09-1.62, p=.004) |
| GIT involved | No | 521 (75.2%) |  |  |
|  | Yes | 172 (24.8%) | 1.13 (0.84-1.52, p=0.427) | 1.03 (0.83-1.29, p=0.772) |
| Oropharynx involved | No | 570 (82.3%) |  |  |
|  | Yes | 123 (17.7%) | 0.95 (0.67-1.36, p=0.786) | 1.04 (0.80-1.34, p=0.789) |
| Exnodal involved | No | 115 (16.6%) |  |  |
|  | Yes | 578 (83.4%) | 3.06 (1.92-4.88, p<.001) | 1.70 (1.28-2.25, p<.001) |
| Year of diagnosis | After 2015 | 456 (65.8%) |  |  |
|  | Before2015 | 237 (34.2%) | 1.65 (1.24-2.20, p<.001) | 1.20 (0.98-1.48, p=.079) |

**SUPPLEMENTAL TABLE 2** Post-treatment Factors Associated with PFS and OS in MCL patients on univariate COX analysis

| **Variable** | | **N (%)** | **OS HR (95%CI,P value)** | **PFS HR (95%CI,P value)** |
| --- | --- | --- | --- | --- |
| Cytarabine | with high-dose cytarabine | 223 (32.2%) |  |  |
|  | without high-dose cytarabine | 470 (67.8%) | 3.33 (2.27-4.90, p<.001) | 1.99 (1.58-2.51, p<.001) |
| Consolidation therapy | Auto-SCT | 80 (11.5%) |  |  |
|  | Chemotherapy | 613 (88.5%) | 2.87 (1.66-4.93, p<.001) | 2.94 (2.00-4.32, p<.001) |
| Maintenance treatment | No | 387 (55.8%) |  |  |
|  | Yes | 306 (44.2%) | 0.25 (0.17-0.35, p<.001) | 0.32 (0.26-0.40, p<.001) |
| Short term efficacy | CR/PR | 587 (84.7%) |  |  |
|  | SD/PD | 106 (15.3%) | 9.74 (7.18-13.21, p<.001) | 10.99 (8.59-14.06, p<.001) |
| Recurrence Refractory | No | 284 (41.0%) |  |  |
|  | Yes | 409 (59.0%) | 10.04 (5.60-17.99, p<.001) | - |

**SUPPLEMENTAL TABLE 3** The proportion of combined and secondary tumors in MCL patients

| **Combined /Secondary tumor** | **N** |
| --- | --- |
| Lung cancer | 7 |
| AML/ALL/MDS/DLBCL | 6 |
| Breast cancer | 4 |
| Prostatic cancer | 3 |
| Gastric cancer | 3 |
| Colon cancer | 3 |
| Rectal cancer | 1 |
| Pancreatic cancer | 1 |
| Nasopharyngeal carcinoma | 1 |
| Renal carcinoma | 1 |
| Liver cancer | 1 |
| Thyroid carcinoma | 1 |
| Pancreatic cancer | 1 |
| Seminoma | 1 |
